# Otologic, audiometric and speech findings in patients undergoing surgery for cleft palate

**DOI:** 10.1186/s12887-018-1312-7

**Published:** 2018-11-08

**Authors:** Cristina Garcia-Vaquero, Cristina Mir, Domingo Graterol, Nuria Ortiz, Maria Isabel Rochera-Villach, Matilde E. LLeonart, Juan Lorente

**Affiliations:** 1 Otolaryngology Department, Vall d’Hebron University Hospital, Passeig Vall d’Hebron 119-129, 08035 Barcelona, Spain; 2 Biomedical Research in Cancer Stem Cells, Vall d’Hebron Research Institute, Passeig Vall d’Hebron 119-129, 08035 Barcelona, Spain

**Keywords:** Cleft palate, Otology, Hearing loss, Audiometry

## Abstract

**Background:**

Although considerable progress has been made in the last 30 years in the treatment of cleft palate (CP), a multidisciplinary approach combining examinations by a paediatrician, maxillofacial surgeon, otolaryngologist and speech and language pathologist followed by surgical operation is still required. In this work, we performed an observational cross-sectional study to determine whether the CP grade or number of ventilation tubes received was associated with tympanic membrane abnormalities, hearing loss or speech outcomes.

**Methods:**

Otologic, audiometric, tympanometric and speech evaluations were performed in a cohort of 121 patients (children > 6 years) who underwent an operation for CP at the Vall d’Hebron Hospital, Barcelona from 2000 to 2014.

**Results:**

The most and least frequent CP types evaluated according to the Veau grade were type III (55.37%) and I (8.26%), respectively. A normal appearance of the membrane was observed in 58% individuals, of whom 55% never underwent ventilation ear tube insertion. No statistically significant associations were identified between the CP type and number of surgeries for insertion of tubes (*p* = 0.820). The degree of hearing loss (*p* = 0.616), maximum impedance (*p* = 0.800) and tympanic membrane abnormalities indicative of chronic otitis media (COM) (*p* = 0.505) among examined patients revealed no statistically significant association with the grade of CP. However, an association was identified between hypernasality and the grade of CP (*p* = 0.053), COM (*p* = 0.000), hearing loss (*p* = 0.000) and number of inserted ventilation tubes.

**Conclusion:**

Although the placement of tympanic ventilation tubes has been accompanied by an increased rate of COM, it is still important to assess whether this is a result of the number of ventilation tubes inserted or it is intrinsic to the natural history of middle ear inflammatory disease of such patients.

Our results do not support improvements in speech, hearing, or tympanic membrane abnormalities with more aggressive management of COM with tympanostomy tubes.

## Background

Clefts of the lip, alveolus and palate are the most common congenital malformations of the head and neck and the second most common of all congenital malformations in general [1]. Both cleft lip and cleft palate (CP) occur when tissues in the face and mouth do not fuse properly by the 2nd or 3rd month of pregnancy. Although considerably progress has been made in the last 30 years in the treatment of CP, multiple surgical procedures are still required followed by endless clinical visits and emotional and physical stress both for patients and their families [2, 3]. The current state of CP treatment relies on a multidisciplinary approach starting from the examination by a paediatrician who oversees the development of the child, a plastic or oral surgeon and an otolaryngologist [4].

Malformations affecting the soft and hard palate influence the functionality of the Eustachian tube, interfering with the ear ventilation and drainage from the ear [5]. Therefore, Eustachian tube dysfunction may lead to middle ear effusion and otitis media recurrence, thus affecting auditory function [6, 7]. The recovery rate of Eustachian tube function after palatal surgery ranges from 40 to 86% [8, 9]. Although the surgical closure of the palate with the pterygoid and tensor of the soft palate muscle integrity may improve middle ear status, the simple closure of the palate is insufficient unless a good velar sphincter is formed [10, 11].

The list of specialists required for the proper treatment of CP is not complete without the identification of ear, nose and throat (ENT) manifestations associated with CP. Follow-up examination involves a maxillofacial surgeon who monitors the development of dentition and palate, an otolaryngologist who evaluates the middle ear status, an audiologist who monitors hearing loss, and a speech and language pathologist. Recommendations for surgical correction and treatment of middle ear pathology are based on otologic and audiometric evaluations. To date, there is still a lack of evidence indicating that a greater degree of alteration of the palate is associated with increased velopalatal and Eustachian tube dysfunction and consequently an increased rate of chronic otitis media (COM) and increased hearing loss [6, 12]. In this regard, understanding Eustachian tube dysfunction is an important component of CP management [13]. Furthermore, the majority of children with CP display acute otitis media at some point in their development. The incidence of acute otitis media in these children is not comparable to that of the general population until adolescence [14].

In the short term, patients treated early with ear tubes exhibit better audiometric results as well as better articulation and speech development compared with patients treated conservatively, whereas no significant differences have been observed regarding mid- or long-term outcomes. Since early placement of tympanic ventilation tubes has been the standard in the care of CP children and often accompanied by an increased rate of tympanic membrane abnormalities indicative of COM, it is important to assess whether this is a result of the insertion of multiple tympanostomy tubes or it is intrinsic to the natural history of middle ear inflammatory disease of such patients [15].

Here, we studied a cohort of CP patients who underwent operation at the Vall d’Hebron Hospital in Barcelona to determine how CP type or degree may affect the presence of tympanic membrane abnormalities indicative of COM among patients who underwent operation for this type of malformation. The impact of CP type and number of ventilation tubes received on otologic, audiologic, and speech outcomes was investigated. Therefore, the state of the ear was reviewed by otoendoscopy/otomicroscopy, and audiometry was performed in external consultations. This information was related to the type of CP not with the type of surgery because we agreed that this variable was uniform in all cases.

## Methods

### Study design

This research is an observational cross-sectional study on a group of patients. Otologic, audiometric, tympanometric and speech evaluation were conducted for a cohort of children (> 6 years) with cleft repair between 2000 and 2014. All individuals were followed by the ENT service of our centre. The type and degree of velopalatal malformation, presence/absence of hearing loss, and number of surgeries for insertion of ear tubes were recorded. The indication used in our service for the placement of the tympanostomy tubes is the presence of persistent seromucosa otitis media (with at least 3 months of evolution) associated with conductive hearing loss greater than 30–35 dB (predominantly in the low frequencies) and/or a delay/alteration in the acquisition of language to a greater or lesser degree.

In addition, patients were classified according to the presence/absence of tympanic membrane abnormalities indicative of COM. The procedures described within the present study are part of routine clinical care.

### Selection criteria

The inclusion criteria were age greater than or equal to 6 years, repair of cleft palate +/− cleft lip between 2000 and 2014, repair performed in our centre, follow-up by ENT in our centre, and complete otoscopic, audiometric, impedance and speech evaluation at the time of the study.

The exclusion criteria were malformative syndrome affecting the facial mass and/or accompanied by alteration of the Central Nervous System, isolated cleft lip, cleft repair at outside hospital, no ENT follow-up, or no otoscopic, audiometric impedance and/or speech testing at the time of this study.

### Otologic measurements

Tympanic membranes were evaluated using microscopic otoscopy. The term chronic otitis media refers to intractable middle ear or mastoid tissue pathology behind an intact or perforated tympanic membrane. The presence of cholesteatoma, deep attic retraction pockets, persistent membrane perforations (> 3 months), atelectasis or severe adhesive membranes were considered as COM. Ears that had a lower degree of shrinkage or persistent otitis media with effusion (> 3 months) without further tympanic alterations were considered abnormal but not as COM. In the same way, the presence of tympanostomy tubes without the presence of other changes in the tympanic membrane, apart from these at the time of examination, was not considered COM. Finally, the presence of isolated myringosclerosis was not considered abnormal, as this finding has not been suggested as a risk factor in the development of COM.

### Audiometric measurements and hearing loss

Pure-tone audiometry was performed on all patients using an audiometer MAICO MA-51 (CE certificate 0124) (Berlin, Germany) and headphones (Telephonics TDH 39-P, New York, USA) properly calibrated in accordance with the manufacturer’s manuals. Five pure tone averages (PTA) at 500, 1000, 2000, 4000, and 8000 Hz were calculated. The degree of hearing loss was evaluated as classified by Northern and Downs [16]. Briefly, 0–15 dB was considered normal hearing, 16–25 dB was considered very mild hearing loss (but included as normal hearing loss during analyses), 26–40 dB was considered mild hearing loss, 41–70 dB was considered moderate hearing loss, 71–90 dB was considered severe hearing loss, and greater than 91 dB was considered profound hearing loss.

### Impedance measurements/tubal evaluation

Middle ear impedance was measured using Titan (Interacoustics, Interacoustics A/S, Middelfart, Denmark).

Type A tympanograms with peak pressures within {plus minus} 50 daPa and compliance of 0.2–1.4 cc were considered normal because it is a normal curve where the pressure in the middle ear corresponds to the atmospheric and therefore to that of the external auditory canal. However, the tympanograms Ad, As, B and C are pathological curves.

### Speech

Speech parameters assessed included nasal resonance and articulation errors. Hypernasality was ranked on a scale of 0–3, which corresponds to the absence of nasal resonance (0), mild (1), moderate (2) and severe (3) hypernasality. Speech articulation was evaluated by demonstrating the presence or absence of errors.

### Statistics

The continuous variables of the study were summarized by descriptive statistics: mean and standard deviation (sd). Similarly, categorical variables were summarized by frequency statistics: number of cases and percentage.

The following relationships were evaluated using Chi-squared or Fisher’s exact test: Veau classification-COM, Veau classification-hypoacusis, Veau classification-abnormal impedance, Veau classification-hypernasality, Veau classification-number of ventilation tube surgeries, number of ventilation tube surgeries-COM, and number of ventilation tube surgeries-hypoacusis.

Multivariate analysis by using the multiple linear regressions was performed to assess the association with Veau classification, age, number of ventilation tubes inserted, degree of hearing loss (independent variables) and COM (dependent variable).

The SPSS program (version 16.0) (Chicago, IL, USA) for data management and the Stata program (version 13.1) (College Station, TX, USA) were used for the remainder of the calculations.

A level of significance α = 0.05 was established in all tests performed.

## Results

### Otoscopic evaluation

**Fig. 1:**
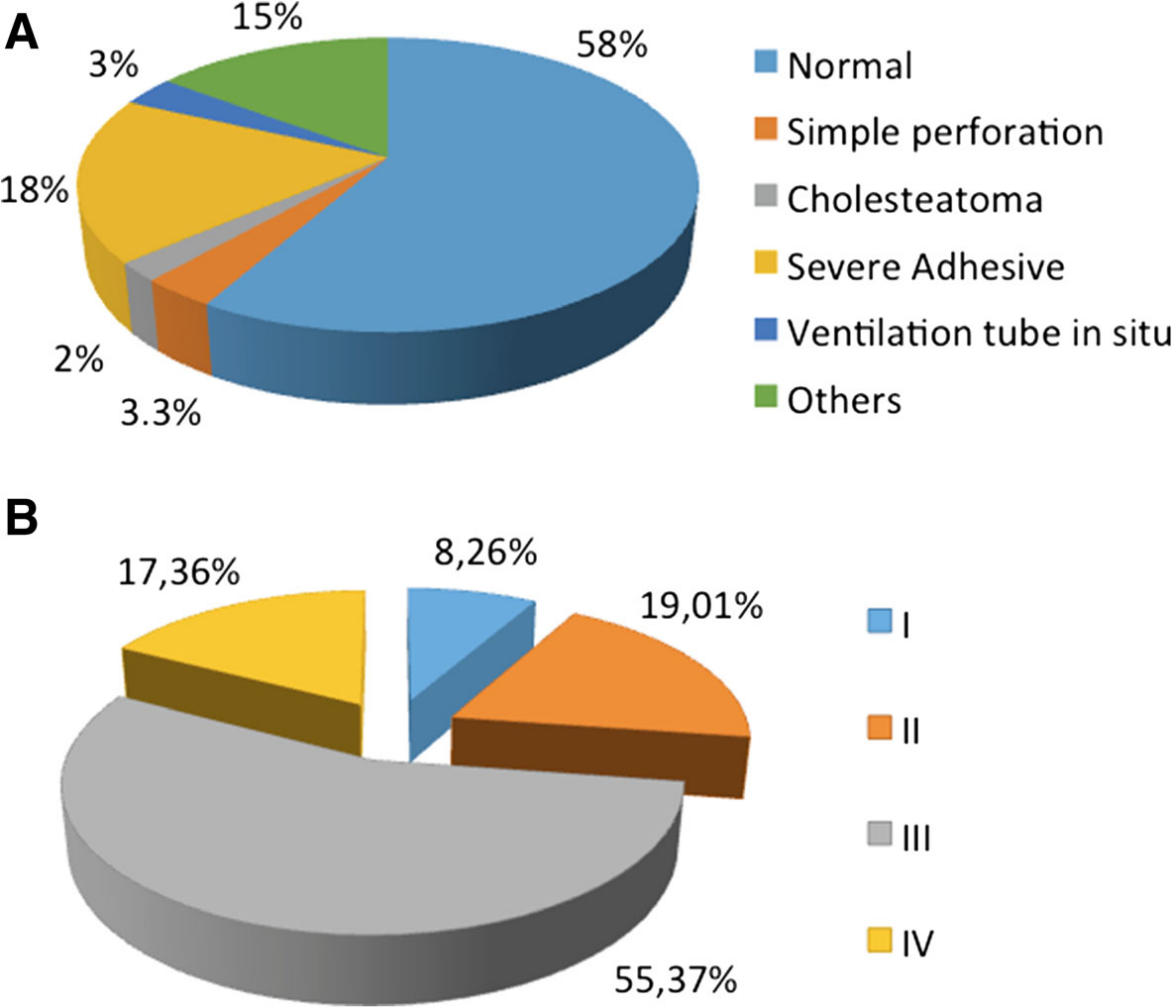
Otoscopic evaluation and cleft palate type. **a** Otoscopy results of 121 individuals (57 female, 64 male) with an age range of 6–31 years. **b** Distribution of patients based on the Veau classification

Otoscopic evaluation was performed on 121 individuals, including 64 males and 57 females. Ages ranged from 6 to 31 years with an average of 13.21 years (sd = 5.94); 50% were older than 12 years. Tympanic membranes were viewed in each of the 242 ears; 58% of the subjects had a normal membrane. Our data revealed 42% individuals with various abnormalities, including severe adhesiveness (18.20%), simple perforation (3.30%), ventilation tubes in situ (3.30%), cholesteatoma (2.10%) and others (monomeric membrane, presence of cavities, or presence of attic pocket) (15%) (Fig. 1a).

### CP analysis

The types of palate were evaluated according to Veau grade [17]. The most frequent palate type was type III, corresponding to the complete unilateral cleft (55.37%). The least frequent palate type was Veau I (8.26%) (Fig. 1b).

No statistically significant association was observed between CP type and the number of surgeries for insertion of ventilation ear tubes (*p* = 0.820) (Table 1). Our data indicated that greater than half of patients with grade III and IV fissures (approximately 48 and 52%, respectively) required insertion of ventilation tubes on at least one occasion, whereas only 30% of patients with grade I and grade II received tubes.

**Table 1:** Association between the number of ventilation tubes and type of cleft palate (*p* = 0.820)

Number of ventilation tubes	Type of cleft palate				Total
	I	II	III	IV	
0	7 (70%)	15 (65.22%)	35 (52.24%)	10 (47.62%)	67 (55.37%)
1	3 (30%)	5 (21.74%)	18 (26.87%)	9 (42.86%)	35 (28.93%)
2	0 (0%)	3 (13.04%)	11 (16.42%)	2 (9.52%)	16 (13.22%)
3	0 (0%)	0 (0%)	2 (2.99%)	0 (0%)	2 (1.65%)
5	0 (0%)	0 (0%)	1 (1.49%)	0 (0%)	1 (0.83%)
Total	10 (8.26%)	23 (19.01%)	67 (55.37%)	21 (17.36%)	121 (100%)

### Analysis of hearing loss, maximum impedance and COM in CP patients

**Fig. 2:**
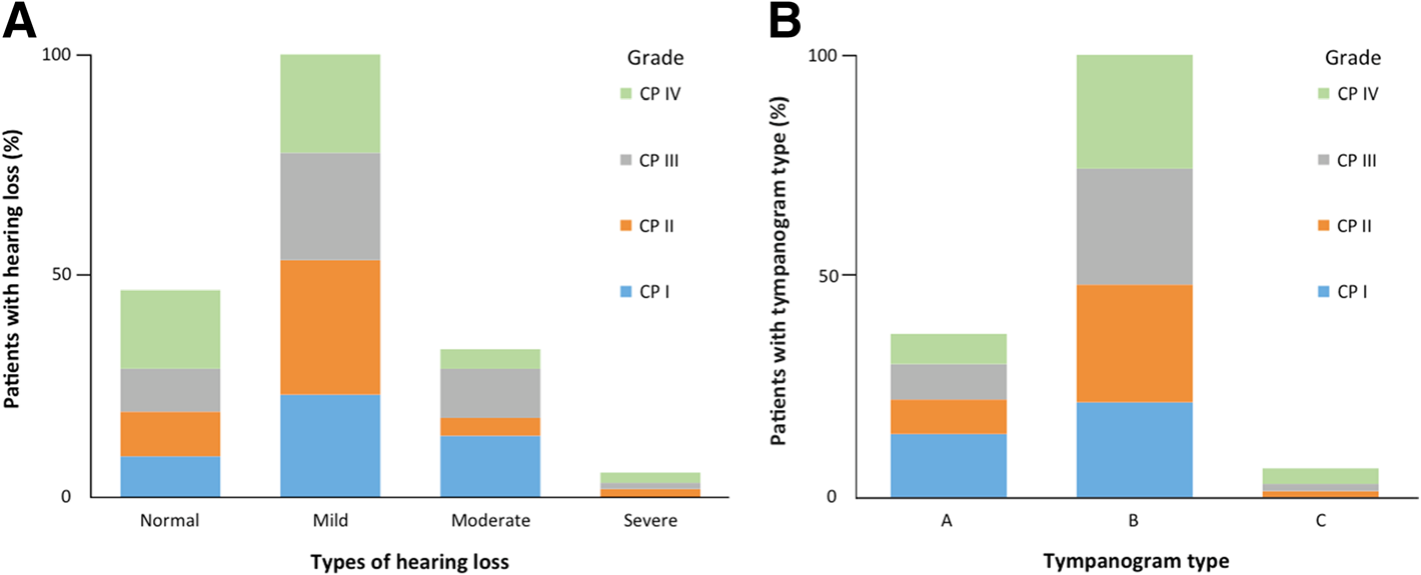
Audiometric measurement of hearing loss in cleft palate (CP) patients. **a** Audiometric analyses were performed to measure hearing loss (%). Data were correlated with the grade of identified CP patients (*p* = 0.616). **b** Middle ear impedance was measured (%) and tympanogram type is represented in relation to the CP type (*p* = 0.800)

We analysed the relationship between the CP type and degree of maximum hearing loss. The degree of hearing loss among examined patients revealed no statistically significant association with the grade of CP (*p* = 0.616) (Fig. 2a). Our data indicated that approximately 23% of patients with grade I, II and III clefts had normal hearing status, whereas 38% of patients with grade IV clefts had normal hearing.

Similarly, Veau classification did not have a significant impact on maximum impedance (*p* = 0.800) (Fig. 2b). Patients with Veau I clefts presented type A curves in 40% of the cases, whereas this proportion decreased to approximately 20% for grade II, III or IV clefts. Similarly, although these results did not imply a difference between the groups, there appeared to be a greater proportion of patients with type C curves in grade II, III and IV malformations (approximately 5%) compared with patients with grade I (0%) fissures. Furthermore, attempts to correlate COM with the CP type and grade revealed no significant association (*p* = 0.505) (Table 2). Although 59.70% of patients with Veau III and 42.86% of patients with Veau IV demonstrated abnormalities consistent with COM, this finding does not support an association between increased cleft complexity and increased probability of COM.

**Table 2:** Association between chronic otitis media (COM) and the type of cleft palate. Here, 121 individuals categorized as COM positive and COM negative were examined based on the grade (from I to IV) of cleft palate (*p* = 0.505)

COM^a^	Type of cleft palate				Total
	I	II	III	IV	
YES	5 (50%)	11 (47.83%)	40 (59.70%)	9 (42.86%)	65 (53.72%)
NO	5 (50%)	12 (52.17%)	27 (40.30%)	12 (57.14%)	56 (46.28%)
Total	10 (8.26%)	23 (19.01%)	67 (55.37%)	21 (17,36%)	121 (100%)

^a^*COM* chronic otitis media

No conclusive or statistically significant results were obtained regarding the relationship of the laterality of the unilateral fissure with the presence of hearing loss, COM or language impairment (data not shown).

### Analysis of hearing loss, COM and ventilation tubes placements

Normal appearance of the membrane was observed in 58% individuals, and a significant portion of these individuals (55%) never underwent ventilation tube insertion (Fig. 2b).

There was a significant association between the number of tubes inserted or hearing loss and COM (*p* = 0.000) (Tables 3 and 4). Normal tympanic membranes were observed in 68.66% of patients with no ventilation tubes compared with 22.86 and 12.50% of those with tubes placed on one or two occasions, respectively (Table 3). The presence of tympanostomy tubes at the time of examination is considered an abnormality because it implies that the tympanic membrane is not integrated. Thus, we were unable to verify the normality of the tympanic membrane. With regard to COM and hearing loss, 82.76% of patients with normal hearing had normal tympanic membranes at the time of examination. Of the 65 patients with COM (53.72%), 39 had mild hearing loss, 18 had moderate hearing loss, and 3 had severe hearing loss. Profound hearing loss was not observed in any individual (Table 4).

**Table 3:** Association between chronic otitis media and the number of ventilation tubes inserted (*p* = 0.000)

COM^a^	Number of ventilation tubes					Total
	0	1	2	3	5	
YES	21 (31.34%)	27 (77.14%)	14 (87.50%)	2 (100%)	1 (100%)	65 (53.72%)
NO	46 (68.66%)	8 (22.86%)	2 (12.50%)	0 (0%)	0 (0%)	56 (46.28%)
Total	67 (55.37%)	35 (28.93%)	16 (13.22%)	2 (1.65%)	1 (0.83%)	121 (100%)

^a^*COM* chronic otitis media

**Table 4:** Association between chronic otitis media (COM) and hearing loss (*p* = 0.000)

COM^a^	Degree of hearing loss				Total
	Normal	Mild	Moderate	Severe	
YES	5 (17.24%)	39 (60%)	18 (78.26%)	3 (75%)	65 (53.72%)
NO	24 (82.76%)	26 (40%)	5 (21.74%)	1 (25%)	56 (46.28%)
Total	29 (23.97%)	65 (53.72%)	23 (19.01%)	4 (3.31%)	121 (100%)

^a^*COM* chronic otitis media

In general terms, the multivariate analysis showed that the presence of COM is more frequently associated with a greater cleft palate type, number of surgeries for the placement of ventilation tubes and degree of hearing loss (Table 5).

**Table 5:** Multivariate analysis showing the association among the grade of cleft palate, age, number of ventilation tubes inserted, hearing loss (independent variables) and chronic otitis media (COM) (dependent variable)

Variable		OR^a^	95% CI^a^	*p*-value
Type of cleft palate	I	1		0.9690
	II	0.73	(0.11; 5.01)	
	III	1.02	(0.19; 5.34)	
	IV	0.90	(0.13; 6.33)	
Age		0.92	(0.84; 1.01)	0.0958
Number of ventilation tubes inserted		8.42	(3.40; 20.89)	0.0000
Hearing loss	Normal	1		0.0013
	Mild	9.88	(2.37; 41.20)	
	Moderate	32.83	(5.61; 191.97)	
	Severe	15.85	(0.66; 379.68)	

^a^*OR* Odds Ratio, *CI* confidence interval

### Analysis of hypernasality of CP patients

One of the most typical characteristics of CP speech is hypernasality [18]. Our data indicated a direct association between hypernasality and CP grade (*p* = 0.053) (Table 6). Up to 48% of patients with grade IV palatine fissures exhibited a moderate degree of hypernasality. Patients with incomplete palatine fissures (grade I) had a 40% absence of nasal resonance at the time of evaluation, whereas this proportion was reduced to approximately 13% (7.46–14.29%) in patients with more complex malformations.

**Table 6:** Association between hypernasality and the cleft palate grade (*p* = 0.053)

Nasality	Type of cleft palate				Total
	I	II	III	IV	
0	4 (40%)	3 (13.04%)	5 (7.46%)	3 (14.29%)	15 (12.4%)
1	5 (50%)	14 (60.87%)	40 (59.7%)	8 (38.1%)	67 (55.37%)
2	1 (10%)	6 (26.09%)	22 (32.84%)	10 (47.62%)	39 (32.23%)
Total	10 (8.26%)	23 (19.01%)	67 (55.37%)	21 (17.36%)	121 (100%)

Overall, our data suggested that the type of cleft malformation did not lead to an increased rate of otologic or audiometric alterations but rather depended on the number of surgeries for insertion of ear tubes.

## Discussion

The association of CP with ear pathology and hearing loss has been studied for greater than a century. CP is believed to be associated with COM of infants and children due to poor Eustachian tube function and other anatomic problems. The current study aimed to determine whether Veau classification or number of ventilation tubes is associated with the presence of COM, hearing loss or speech alteration.

Despite the results obtained, we cannot be sure whether the positive relationship between COM and the number of surgeries is related to surgery or with the natural course of a more aggressive ear pathology that makes it necessary to place a greater number of ventilation tubes in this type of patient. However, we can confirm that those patients with tympanic and/or middle ear alterations present worse hearing compared with patients with a relatively normal otoscopy. In a previous study, the rate of type A and B tympanograms, the proportion of discharge during myringotomy, and the indwelling period of the first ventilating tube insertion also influenced the probability of additional ventilation tube insertion in CP patients [19].

Little information is known about the natural history of CP patients who have not been subjected to palatoplasty or any intervention at the otologic level. Based on the vocal quality characteristics of children with CP, a comprehensive speech and language pathology intervention is recommended [20]. However, one of the earlier works on Dutch CP patients demonstrated only small differences between speech pathologists with and without expertise in CP speech [21]. Studies performed in India and China on patients with unrepaired CP revealed that a significant percentage of these patients demonstrated hearing loss and abnormal tympanometry that persisted in adolescence and adulthood [22]. Another audiological study in Turkey revealed that the prevalence of middle ear disease of children whose palates were repaired during the first two years of life and who did not have access to otolaryngology care was considerably reduced compared with patients who did not receive an operation for palatoplasty or treated by the otorhinolaryngologist. This comparative data analysis demonstrated that palate repair is an independent variable in reducing the prevalence of middle ear pathology [23]. Although there is no definite evidence that a specific CP repair technique results in superior middle ear function, one previous study demonstrated that patients who underwent Furlow Z-plasty required fewer tympanostomy tubes compared with those who underwent traditional techniques [24].

The type of palatine defect plays an important role in the development of mucosal otitis media (OM) and has been observed in patients with major defects requiring additional surgeries for the insertion of tympanostomy tubes [25]. It is not universally accepted that an early and successful closure of the palate reduces the possibility of permanent otological pathology. The initial hypothesis for the early (up to 4 months) closure of the palate that prevents middle ear complications in CP patients is no longer supported. It seems to be clear is that an improvement in hearing is associated with child growth with minimal loss observed after 10 years of age. The variability in the time and course of this recovery suggests that the improvement is not exclusively the result of palatal surgery but a combination of factors, including surgical correction, developmental factors and treatment of middle ear pathology [22].

The number of tympanostomy tubes inserted appears to be related to the abnormal mid-term and long-term tympanic findings. However, atrophy, retraction, and COM may also appear as sequelae in children with persistent mucosal OM who never had tympanostomy tubes [26]. The long-term consequences of drainage insertion are particularly important in CP children. An increased rate of abnormal tympanic membranes between ears previously treated with ventilation tubes in children with CP who underwent operation was previously observed by Ovesen and Belgvad-Andersen [27], and an increased incidence of complications and worse audiological status appears in the studies performed by Robson et al. [28]. However, it is unclear whether children with early placed ventilation tubes later developed mucosal OM.

Focal atrophy and tympanosclerosis are two well-documented types of complications of ventilation tube insertion. The tympanic membrane with tympanosclerosis is considered in numerous abnormal studies despite its small tendency to progress to COM with a minimal effect on hearing. Persistent perforations (9.13%) may also occur, particularly after insertions of repeated tubes, interventions at very early age, or the insertion of permanent drainage tubes, such as Goode’s T-tubes [29].

Cholesteatoma appears in studies as a complication of ventilation tube insertion, especially after insertion of T-tubes. The relationship between COM and cholesteatoma is clear in CP patients, with rates ranging from 0.90 to 9.20% for cholesteatoma. In our case, the frequency of cholesteatomatous COM was 2.06% as it was only observed in 5 patients with this type of pathology. The incidence of COM in this type of patients is variable (11–37%). Persistent tympanic perforation (1.30–19%), tympanic retraction (11.50–36.80%), tympanic sclerosis (11–37%), secondary cholesteatoma (0–3.80%) and otorrhea (up to 11.50%) present with great variability based on the revised study. In our study, we exclusively focused on membrane changes, such as retraction and ‘pexia’, that could be considered to be risk factors for the possible progression to COM when tympanosclerosis is not considered to be an abnormal appearance of the tympanic membrane.

The involvement of COM in CP remains debated. A recent study on 105 CP Turkish patients demonstrates a statistically significant relationship between the middle ear disease, function of Eustachian tube, treatment outcomes, middle ear pressure, CP type and bilateralism of the disease [30]. A study from the Nova Scotia Hearing Center proposed COM with effusion following CP repair as a clinical predictor for secondary velopharyngeal insufficiency [31]. A recommendation for the outcomes that should be measured has been recently proposed by a group of UK clinics performing management of COM in children with CP [14].

In our study, we observed no statistical association between CP type and COM. Accordingly, no statistically significant relationship was noted between palate type and COM (*p* = 0.546), the need for an increased number of ventilation tubes inserted (*p* = 0.820) and grade of hearing loss (*p* = 0.616). Although these results did not imply a difference between the groups, patients with a grade III or IV Veau fissure exhibit an increased frequency of COM (59 and 42.86%, respectively) and a greater degree of hearing loss (up to 38% of patients with grade IV fissures had hearing loss). Similarly, patients with more complex palatine fissures (grades III and IV) require a greater number of surgeries for insertion of ventilation tubes (approximately 50% of patients compared with 30% for patients with Veau grade I or II). Given the lack of significance of the results obtained, we conclude that further studies would be necessary to determine whether this finding was influenced by sample size or whether the relationship described was not significant.

According to our data, ears with an abnormal otoscopic appearance were almost identical to those with a greater number of tubes. A significant correlation was noted between abnormal otoscopy and reduced hearing. The association between the number of inserted ventilation tubes and the increased prevalence of abnormal otoscopy and hearing has been reported. However, it was impossible to determine to what degree the finding is due to the number of surgeries and to what extent the nature of a more severe inflammatory process makes it necessary to place more drainage tubes. It is important to note that our study does not contemplate those patients who have been treated at other centres, some of whom may have developed otological problems. This fact may lead to over- or underestimation of the presence of COM in CP patients who underwent operation. Moreover, it is possible that the children included in our study have developed COM since the end of the study period.

Hearing loss is a known complication of CP patients, but its magnitude is not always appreciated. The majority of the studies focus on the development of a competent velopharyngeal sphincter and normal facial development in this type of patient, while the complication of hearing loss is neglected [32]. Some evidence revealed that children growing with CP exhibited an improved audiological status. Accordingly the drainage can correct the auditory deficit associated with short-term mucosal OM [6, 7].

Recent studies on hearing loss among CP infants revealed no significant difference in patients with or without CP [33]. Another study examining the association among CP grade, palate repair technique, and hearing outcomes in children from northern Finland aged 3–9 years revealed that only 3.30% of CP patients exhibited abnormal hearing [34]. Our analyses of the audiometric and otologic measurements made for the group of CP patients who underwent operation at the Vall d’Hebron Hospital (Barcelona, Spain) revealed no significant association between hearing loss and Veau classification or Eustachian tube function (Table 3b).

In our study, most ears examined (*n* = 242) exhibited mild hearing loss (49.17%). In addition, 34.30% of the ears evaluated exhibited normal hearing (0–25 dB), 14.04% had moderate hearing loss and only 2.47% of the ears had severe hearing loss. Profound hearing loss was not noted in any patient. It seems likely that patients who received a greater number of ventilation tubes displayed an increased rate of COM and worse audiometric results in the long term. Up to 86% of the patients in our study who had COM underwent operation for at least two occasions of transtympanic drainage. Only 17.24% of these patients had strictly normal hearing.

## Conclusions

Our results demonstrate that the CP type did not affect articulation, speech development or hearing loss and was also not associated with COM. By contrast, these parameters seemed to vary depending on the number of tympanostomy tubes inserted. These results do not support improvements in speech, hearing, or tympanic membrane abnormalities indicative of COM with tympanostomy tubes. Although the placement of tympanic ventilation tubes has been often accompanied by an increased rate of COM, it is important to assess whether this is a result of the number of ventilation tubes inserted or it is intrinsic to the natural history of middle ear inflammatory disease of such patients, so more studies are needed.

In the case of children, CP is believed to be associated with COM due to poor Eustachian tube function and other anatomic problems, so understanding Eustachian tube dysfunction is an important component of CP management.

There are many reasons to suggest that mucous OM in these children should be treated conservatively in early childhood. Given that conservative management of mucosal OM is relatively safe and has no long-term sequelae and that other forms of auditory rehabilitation, such as a hearing aid, are available, clinicians could consider more conservative management in light of these data; however, more studies are needed. Nevertheless, the improvement is not exclusively the result of palatal surgery but a combination of factors, including surgical correction, developmental factors, treatment of middle ear pathology and a multidisciplinary approach.

## Abbreviations

COM: Chronic Otitis Media
CP: Cleft palate
ENT: Ear, nose and throat
OM: Otitis media
PTA: Pure tone averages
Sd: Standard deviation
